# CD4^+^CD8^+^ double-positive T cells are associated with severity of tuberculosis

**DOI:** 10.3389/fcimb.2026.1887199

**Published:** 2026-08-25

**Authors:** Bingfen Yang, Hongjuan An, Pei Zhao, Zhihong Cao, Jinwen Su, Xiaoou Wang, Yinping Liu, Xinjing Wang, Jing Jiang, Xiaoxing Cheng

**Affiliations:** 1 Beijing Key Laboratory of New Techniques of Tuberculosis Diagnosis and Treatment, Institute of Tuberculosis Research, Senior Department of Tuberculosis, the Eighth Medical Center of Chinese People's Liberation Army (PLA) General Hospital, Beijing, China; 2 Tuberculosis Control Team, Senior Department of Tuberculosis, The Eighth Medical Center of PLA General Hospital, Beijing, China; 3 Outpatient Department, Senior Department of Tuberculosis, the Eighth Medical Center of PLA General Hospital, Beijing, China; 4 Intensive Care Unit, Senior Department of Tuberculosis, the Eighth Medical Center of PLA General Hospital, Beijing, China; 5 Institute of Research, Beijing Key Laboratory of Organ Transplantation and Immune Regulation, Senior Department of Respiratory and Critical Care Medicine, the Eighth Medical Center of PLA General Hospital, Beijing, China

**Keywords:** DP T cells, IFN-γ, immune cells, Mendelian randomization, tuberculosis

## Abstract

**Background:**

Tuberculosis (TB) remains a global public health burden, and how immune cell subsets regulate host anti-TB immunity and disease progression remains incompletely understood. While previous studies have focused on single-positive (SP) T cells (CD4^+^ or CD8^+^) in TB pathogenesis, the association between CD4^+^CD8^+^ double-positive (DP) T cells and TB susceptibility, severity, and treatment outcomes have not been fully elucidated. This study aimed to investigate the relationship between DP T cells and other immune cell subsets with TB, and to explore the potential diagnostic and prognostic value of DP T cells in active TB.

**Methods:**

A Genome-Wide Association Study (GWAS) was conducted to analyze 731 immune cell traits and a dataset encompassing 895 patients with TB. Subsequently, a cohort including 647 patients with active TB and 632 healthy controls was used to verify the findings of Mendelian randomization (MR). The correlation between the percentage of DP T cells in lymphocytes and TB severity, treatment efficacy, and *Mycobacterium tuberculosis* (Mtb)-specific IFN-γ production was evaluated. Finally, a random forest model incorporating the percentage of DP T cells in leukocytes and other peripheral blood parameters was constructed to distinguish severe from mild active TB.

**Results:**

MR analysis suggested potential causal links between the percentage of DP T cells among peripheral leukocytes and TB status. Clinical sample validation showed that the percentage of peripheral DP T cell among leukocytes was significantly lower in patients with active TB than in healthy controls (P < 0.001), and was inversely correlated with disease severity. Additionally, the percentage of DP T cells in leukocytes was positively correlated with Mtb-specific antigen-stimulated IFN-γ production. Flow cytometric analysis demonstrated that DP T cells had a significantly higher frequency of IFN-γ-expressing cells compared to CD8^+^ SP T cells (P < 0.001). The constructed random forest model effectively distinguished severe from mild TB, with good diagnostic performance (AUC = 0.985).

**Conclusions:**

Our findings indicate that DP T cells are closely associated with TB severity, and are positively associated with Mtb-specific IFN-γ response. The percentage of peripheral DP T cells in leukocytes could serve as a potential non-invasive biomarker for TB severity stratification.

## 1 Introduction

Tuberculosis (TB), an ancient infectious disease caused by *Mycobacteriumtuberculosis* (Mtb), continues to threaten global public health. The latest WHO Globaltuberculosis report estimates that 10.7 million new cases emerging worldwide, leading to 1.23 million deaths from this infectious disease. Despite advancements in diagnostics and therapeutics, TB remains a threat to human health, particularly in low- and middle-income countries where the burden is most heavy (WHO, 2025).

A key challenge in TB control is understanding the mechanisms underlying the progression from latent Mtb infection (LTBI) to active disease: approximately a quarter of the global population is latently infected, yet only 5-10% develop active TB. This disparity highlights the critical role of host innate and adaptive immunity in regulating TB pathogenesis, making immune-related factors a central focus of TB research (Menzies et al., 2018; Chandra et al., 2022). Immune cells are the frontline defenders against Mtb infection, and their dysregulation is closely linked to the transition from LTBI to active TB. Among peripheral immune cells, which circulate in the bloodstream and migrate to infection sites, T cell subsets have been increasingly recognized as potential predictive biomarkers for TB susceptibility and disease progression (Berry et al., 2010; Zak et al., 2016; Cai et al., 2020). However, most previous studies have focused on classical CD4^+^ or CD8^+^ single-positive (SP) T cells, while the role of CD4^+^CD8^+^ double-positive (DP) T cells in TB immunity remains poorly understood. Given the complex interplay between host immunity and Mtb, identifying immune cell subsets with causal relevance to TB risk and their functional roles could provide novel insights into TB pathogenesis and inform the development of targeted biomarkers or immunotherapies.

Traditional observational studies often face limitations such as confounding biases, making it difficult to establish causal relationships between immune traits and TB. Mendelian randomization is an innovative epidemiological approach that uses Single Nucleotide Polymorphisms (SNPs) as instrumental variables to infer causality, overcoming the limitations of observational studies when randomized controlled trials are infeasible or unethical (Burgess et al., 2023; Skrivankova et al., 2021). Genome-Wide Association Studies (GWAS) have successfully identified genetic variants associated with peripheral immune cell traits, providing a valuable resource for MR analyses to explore causal links between immune phenotypes and diseases (Orru et al., 2020). However, no studies have integrated GWAS and MR to investigate the causal relationship between peripheral immune cell subsets and TB risk.

Herein, we aimed to fill this research gap by: (1) performing MR analysis using GWAS data on 731 immune cell traits to identify subsets causally associated with TB; (2) validating the key findings in a clinical cohort of active TB patients and healthy controls; and (3) exploring the functional relevance of the identified immune cell subset to TB severity, treatment efficacy, and Mtb-specific immune responses. This study provides a novel genetic and clinical perspective on the role of immune cell subsets in TB, with implications for the development of improved risk stratification tools and host-directed therapeutic strategies.

## 2 Materials and methods

### 2.1 Mendelian randomization

A two-sample Mendelian randomization approach was employed to investigate the causal relationship between immune cell traits and TB. Instrumental variables (IVs) were selected based on three core assumptions: relevance (IVs strongly associated with immune cell traits), independence (IVs unrelated to confounders), and exclusion restriction (IVs affecting TB only through immune cell traits) (Skrivankova et al., 2021). GWAS summary statistics for immune cell traits were retrieved from the IEU Open GWAS Project, with accession numbers ranging from ieu-a-GCST90001391 to ieu-a-GCST90002121. This dataset encompasses 3757 samples, more than 20 million single nucleotide polymorphisms (SNPs), and covered 731 distinct immune cell traits (Orru et al., 2020). GWAS data for TB were also obtained from the IEU Open GWAS Project (accession: ieu-a-GCST90018892), including 477386 individuals (895 TB cases and 476,491 controls) and 24189689 genotyped SNPs (Sakaue et al., 2021). Since both exposure and outcome GWAS rely on European-ancestry samples, the genetic causal estimates generated in this MR analysis cannot be directly generalized to East Asian TB cohorts. Nevertheless, this large-scale MR screening still provides robust genetic evidence to rule out lifelong genetically determined peripheral immune profiles as intrinsic predisposing factors for TB infection in European populations.

Strict criteria were applied to select qualified IVs for MR analysis. SNPs associated with immune cell traits were included at a threshold of p < 1×10^-5^, with an F-statistic > 10 to ensure adequate statistical power. To mitigate linkage disequilibrium, SNPs were clumped at r² < 0.001 within a 1000 kb window. The LDlink database was used to control for potential confounders, including smoking status, alcohol consumption, anemia, HIV infection, body weight, body mass index, lung function, asthma, diabetes, metabolic disorders, obesity, insomnia, silicosis, chronic kidney disease, and glomerular filtration rate (Hamilton et al., 2025; Obore et al., 2020; Dai et al., 2019).

All MR analyses were performed using the R package TwoSampleMR (v0.6.6). Five complementary MR methods were adopted to verify result robustness: inverse variance weighting (IVW), weighted median, MR-Egger regression, simple mode, and weighted mode regression. A p-value < 0.05 was defined as statistically significant. Scatter plots were generated to visualize the SNP-trait association patterns.

Cochran’s Q statistic (for IVW) and Rücker’s Q statistic (for MR-Egger) were calculated to assess between-SNP heterogeneity. Horizontal pleiotropy was evaluated using the MR-PRESSO test and the MR-Egger regression intercept. A p-value > 0.05 indicated no significant heterogeneity or horizontal pleiotropy. Leave-one-out sensitivity analysis was further conducted to confirm the reliability of the causal estimates.

### 2.2 Patients and healthy controls

Participants with active TB were enrolled from hospitalized patients admitted to the Department of Tuberculosis, Eighth Medical Center of PLA General Hospital, between January 1, 2023 and December 31, 2024. All active TB cases were diagnosed in accordance with the national standard WS 288-2017: Diagnostic Criteria for Pulmonary Tuberculosis (2017 edition) issued by the National Health and Family Planning Commission of China (National Health Commission of the People's Republic of China., 2017). Age- and sex-matched healthy individuals undergoing routine annual physical examination without any clinical or imaging evidence of TB were recruited as healthy controls.

Exclusion criteria included a history of malignancy, allergic diseases, immunocompromised status (e.g., organ transplantation, systemic lupus erythematosus, ankylosing spondylitis, rheumatoid arthritis), and other infectious conditions such as HBV, HCV, HIV, and COVID-19 infection.

Clinical and laboratory data, including clinical symptoms, sputum smear, Mtb culture, Xpert detection, and radiological findings, were retrospectively collected from electronic medical records (**Table 1**).

**Table 1:** Demographic and clinical characteristics of patients with TB and controls.

Characteristic	TB patients (*n* = 647)	Healthy controls (*n* = 632)
Sex (female/male)	447/200	454/178
Age (mean ± SD)	39.59 ± 15.77	41.12 ± 15.22
Pathogen (+/–)	429/218	NA^a^
AFB (+/-/NA)	119/432/96	NA
Culture (+/-/NA)	144/326/177	NA
TB-DNA (+/-/NA)	178/316/153	NA
Xpert (+/-/NA)	289/254/104	NA
IGRA (+/–/NA)	360/84/203	82/166/384
Dissemination (1/2/3/4)^b^	180/336/100/29	NA
PTB/EPTB/PTB&EPTB	482/91/73	NA
Severe TB (+/-)^c^	67/580	NA
Therapy effect (good/poor/death)^d^	363/264/20	NA
Diabetes (+/-)	97/550	NA

^a^Not available. ^b^Extent of TB lesions: 1, Lesions confined to one organ; 2, Lesions involving two organs; 3, Lesions involving three organs; 4, Lesions involving four or more organs. ^c^Disease severity: +, Severe TB: requiring invasive mechanical ventilation or complicated by one or multiple organ failures; −, Mild TB: no requirement for invasive mechanical ventilation and no organ failure. ^d^Treatment outcome: good, sputum smear or culture conversion to negative, or obvious lesion absorption within one month of anti-TB treatment; poor, persistently positive sputum smear or culture, or no lesion absorption with even enlarged lesions after one month of treatment; death, in-hospital death within one month after sample collection.

Peripheral blood samples for immune detection were collected within 3 days after patients began inpatient anti-TB chemotherapy. All patients had active TB at sampling, and several individuals had prior anti-TB treatment history before admission.

Mild and severe TB subgroups were stratified according to the primary diagnostic indicators laid out in the “Guidelines for definition and diagnosis of severe pulmonary tuberculosis in adults in China (2023)” (Tuberculosis Professional Committee of Chinese Research Hospital Association et al., 2024), a national clinical practice guideline tailored for Chinese patients with TB. Severe TB was defined as patients requiring invasive mechanical ventilation or complicated by single or multiple organ failure, while patients without invasive ventilation demands and organ impairment were classified as mild TB. We only adopted primary diagnostic indicators for severity stratification, as consistent, complete retrospective documentation of all secondary clinical, laboratory and radiological indicators could not be reliably obtained across all enrolled patients.

### 2.3 Peripheral blood lymphocyte subset detection

Peripheral blood samples were added to an absolute counting tube (Cat. No. 662995, BD Biosciences), followed by the addition of 20 μL lymphocyte subset reagent containing fluorescent-labeled antibodies against CD3, CD8, CD45, CD4, CD16, CD56, and CD19 (Cat. No. 662967, BD Biosciences). The mixture was incubated at room temperature in the dark for 15 min. Subsequently, 450 μL of diluted lysing solution (Cat. No. 349202, BD Biosciences) was added, and the samples were incubated for an additional 15 min under the same conditions. Finally, the percentages and absolute counts of peripheral lymphocyte subsets were acquired using a BD FACSCanto II flow cytometer and analyzed with BD FACSCanto software.

### 2.4 Plasma cytokine detection

Plasma cytokine concentrations were quantified using a multiplex flow cytometric immunofluorescence assay (Cat. No. R701002, Raisecare, China) following the manufacturer’s instructions. Briefly, capture microspheres were mixed with plasma samples and incubated at room temperature for 2 h in the dark with shaking at 300 rpm. Phycoerythrin-labeled streptavidin was then added, followed by a further 30 min incubation. After washing with diluted wash buffer, fluorescence intensity in calibration and sample tubes was detected by flow cytometry. Plasma cytokine levels were calculated using LEGENDplex v8.0 software.

### 2.5 TB antigen-specific interferon-γ release assay

Antigen-specific interferon-γ release assay was performed using a Chemiluminescent Microparticle Immunoassay kit for TB-specific T-cell detection (innoDx, China). Briefly, 4 mL fresh peripheral whole blood was collected into heparin-anticoagulated tubes. Within 16 h after collection, 1 mL whole blood was transferred into three tubes: a test tube containing recombinant Mtb antigens, a negative control tube, and a positive control tube supplemented with PHA. All samples were incubated at 37 °C in a cell culture incubator for 22 ± 2 h. Subsequently, plasma was separated by centrifugation at 3000 rpm for 10 min.

Then, 50 μL plasma from each tube and serially diluted standard samples were added to an ELISA microplate pre-coated with anti-interferon-γ antibody, followed by incubation at 37 °C for 60 min. After incubation, 50 μL enzyme-conjugated reagent was added, and the plate was incubated again at 37 °C for another 60 min. Following plate washing, 50 μL of chromogenic solutions A and B were added sequentially, and the color reaction was developed at 37 °C for 15 min. The reaction was terminated by adding 50 μL stop solution to each well. The absorbance at 450 nm was measured using an ELISA reader within 10 min. A standard curve was generated based on the optical density values of the standard series, and plasma IFN-γ concentrations were calculated accordingly.

### 2.6 Intracellular staining

Intracellular staining was performed as previously described (Jiang et al., 2024). Briefly, isolated peripheral blood mononuclear cells (PBMCs) were resuspended in complete RPMI 1640 medium (GIBCO) and stimulated with CD3/CD28 monoclonal antibody-coated beads (Dynabeads Human T-Activator CD3/CD28, GIBCO) for approximately 20 h.

After stimulation, PBMCs were washed and surface-stained with fluorochrome-conjugated antibodies: CD3/APC-H7 (clone SK7), CD8/Alexa Fluor 647 (clone RPA-T8) from BD Biosciences, and CD4/Alexa Fluor 700 (clone OKT4) from BioLegend.

Cells were fixed and permeabilized using the Foxp3 Transcription Factor Staining Buffer Set (eBioscience, Thermo Fisher Scientific), followed by intracellular staining with IFN-γ/Brilliant Violet 510 (clone 4S.B3, BioLegend). Finally, cells were acquired and analyzed on a BD FACSCanto II flow cytometer (BD Biosciences).

### 2.7 Model construction and validation

Random forest (RF) prediction models were established based on seven peripheral blood biomarkers with AUC values greater than 0.65. These biomarkers included the percentages of DP T cells, lymphocytes, and monocytes in leukocytes, plasma IL-6 concentration, as well as absolute counts of lymphocytes, NK cells, and T cells. Model hyperparameters were optimized using 5-fold cross-validation with stratified sampling to divide the dataset into training and internal validation sets. The predictive performance of each RF model was assessed using the area under the receiver operating characteristic curve (AUC).

### 2.8 Statistical analysis

Mann-Whitney test was used for statistical analysis between two groups, Kruskal-Wallis test with Dunn’s multiple comparisons test was used for statistical analysis between three or more groups. Pearson correlation was used for correlation analysis. The effect size of difference was shown as Cliff’s δ for Mann-Whitney test, if |δ| < 0.147, it indicates the effect is so small that it can be ignored; if 0.147 ≤ |δ| < 0.33, it indicates a small effect; if 0.33 ≤ |δ| < 0.474, it indicates a medium effect; if |δ| ≥ 0.474, it indicates a large effect. All statistical tests were performed with GraphPad Prism 6.0 (USA) or the R package “correlation”, “effsize” and “pROC”. Statistical tests were two tailed, and p<0.05 was considered significant.

## 3 Results

### 3.1 Causal relation of immunophenotypes on TB

To screen genetically regulated immune biomarkers correlated with TB susceptibility, we performed a comprehensive two-sample Mendelian randomization analysis covering 731 immune cell phenotypes. All genetic summary statistics were obtained from the IEU Open GWAS Project database, with accession numbers ranging from ieu-a-GCST90001391 to ieu-a-GCST90002121. These data cover various types of immune cells, including B cells, T cells, natural killer cells, Treg cells, and their related parameters and proportions. A European population dataset containing 3757 samples and 22 million single nucleotide polymorphisms (SNPs) was used. All genetic effect estimates generated in the present MR analysis were derived from European-ancestry GWAS cohorts. Owing to distinct genetic architectures across ethnic groups, the observed genetic associations cannot be directly generalized to East Asian patients with TB.

**Figure 1:**
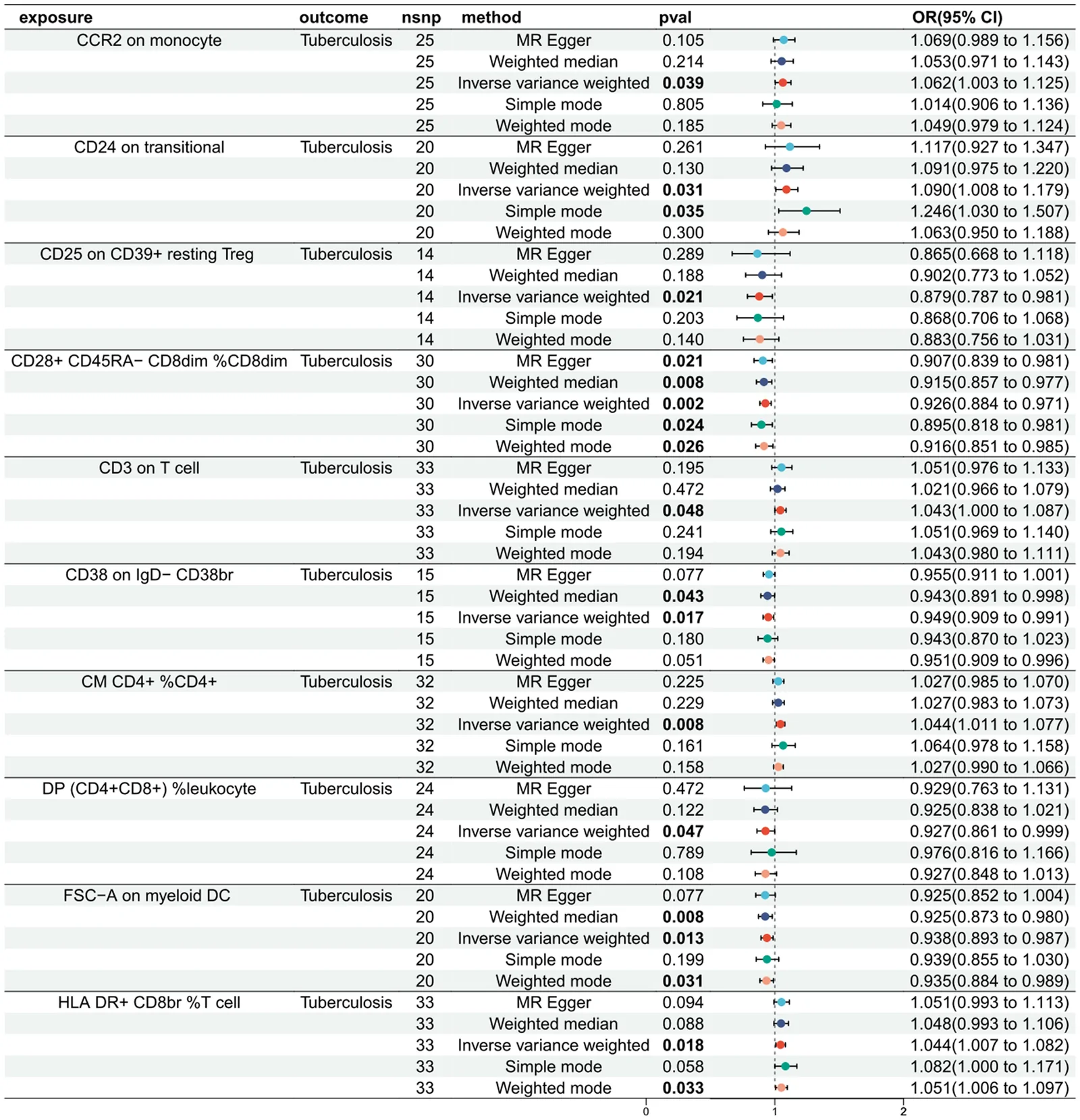
Forest plots of the results of MR analysis of the causal relationship between immune cell traits and TB. CCR2 on monocytes: the MFI (mean fluorescence intensity) of CCR2 on monocytes; CD24 on transitional: the MFI of CD24 on transitional (CD24^+^CD38^high^) B cells; CD25 on CD39+ resting Treg: the MFI of CD25 on CD39^+^ resting (CD25^++^CD45RA^+^) Treg; CD28+ CD45RA-CD8dim %CD8dim: the percentage of CD28^+^CD45RA^-^CD8^dim^ cells in CD8^dim^ cells; CD3 on T cell: the MFI of CD3 on T cells; CD38 on IgD-CD38br: the MFI of CD38 on IgD^-^CD38^bright^ B cells; CM CD4+ %CD4+: the percentage of CD4^+^ T_CM_ (central memory, CCR7^+^CD45RA^-^) in CD4^+^ T cells; DP (CD4+CD8+) %leukocyte: the percentage of CD4^+^CD8^+^ DP T cells in leukocytes; FSC-A on myeloid DC: FSC-A on myeloid DCs; HLA DR+ CD8br %T cell: the percentage of HLA-DR^+^CD8^bright^ cells in T cells.

**Figure 2:**
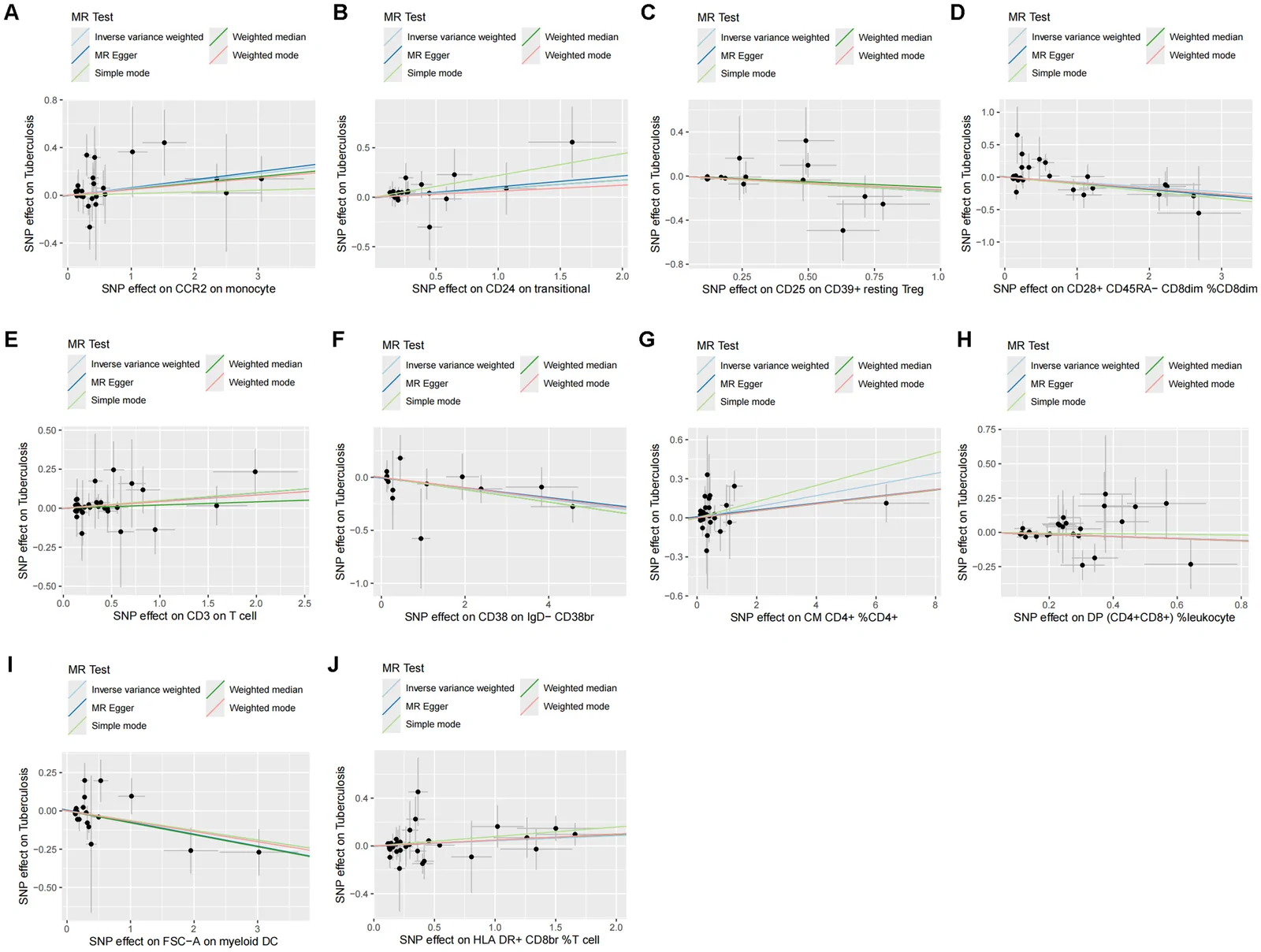
Scatter plots of the results of MR analysis of the causal relationship between immune cell traits and TB. **(A)** The MFI of CCR2 on monocytes; **(B)** The MFI of CD24 on transitional (CD24⁺CD38^high^) B cells; **(C)** The MFI of CD25 on CD39⁺ resting (CD25⁺⁺CD45RA⁺) Treg; **(D)** The percentage of CD28⁺CD45RA⁻CD8^dim^ cells in CD8^dim^ cells; **(E)** The MFI of CD3 on T cells; **(F)** The MFI of CD38 on IgD⁻CD38^bright^ B cells; **(G)** The percentage of CD4⁺ T_CM_ (central memory, CCR7⁺CD45RA⁻) in CD4⁺ T cells; **(H)** The percentage of CD4⁺CD8⁺ DP T cells in leukocytes; **(I)** FSC-A on myeloid DCs; **(J)** The percentage of HLA-DR⁺CD8^bright^ cells in T cells.

Bonferroni correction was applied to control type I error across 731 independent tests, with a rigorous significance threshold set at 0.05/731 ≈ 6.84×10^-5^. Raw IVW P < 0.05 was defined as only nominally suggestive association for exploratory candidate filtering, yet none of the traits with unadjusted P < 0.05 met the Bonferroni threshold for robust germline genetic causality. At this nominal unadjusted P-value, 10 immune phenotypes displayed suggestive correlations with TB (**Figures 1**, **2**). Among them, five immune cell traits were risk factors for TB: the percentage of HLA-DR^+^CD8^bright^ cells in T cells (OR (95%CI) = 1.044 (1.007 to 1.082), p = 0.018); the percentage of CD4^+^ T_CM_ (central memory, CCR7^+^CD45RA^-^) in CD4^+^ T cells (OR (95%CI) = 1.044 (1.011 to 1.077), p = 0.008); the MFI (mean fluorescence intensity) of CD3 on T cells (OR (95%CI) = 1.043 (1.000 to 1.087), p = 0.048); the MFI of CD24 on transitional (CD24^+^CD38^high^) B cells (OR (95%CI) = 1.090 (1.008 to 1.179), p = 0.031); the MFI of CCR2 on monocytes (OR (95%CI) = 1.062 (1.003 to 1.125), p = 0.039).

There were 5 immune cell traits protecting against TB: the percentage of CD4^+^CD8^+^ DP T cells in leukocytes (OR (95%CI) = 0.927 (0.861 to 0.999), p = 0.047); the percentage of CD28^+^CD45RA^-^CD8^dim^ cells in CD8^dim^ cells (OR (95%CI) = 0.926 (0.884 to 0.971), p = 0.002); the MFI of CD25 on CD39^+^ resting (CD25^++^CD45RA^+^) Treg (OR (95%CI) = 0.879 (0.787 to 0.981), p = 0.021); the MFI of CD38 on IgD^-^CD38^bright^ B cells(OR (95%CI) = 0.949 (0.909 to 0.991), p = 0.017); FSC-A on myeloid DCs (OR (95%CI) = 0.938 (0.893 to 0.987), p = 0.013).

**Figure 3:**
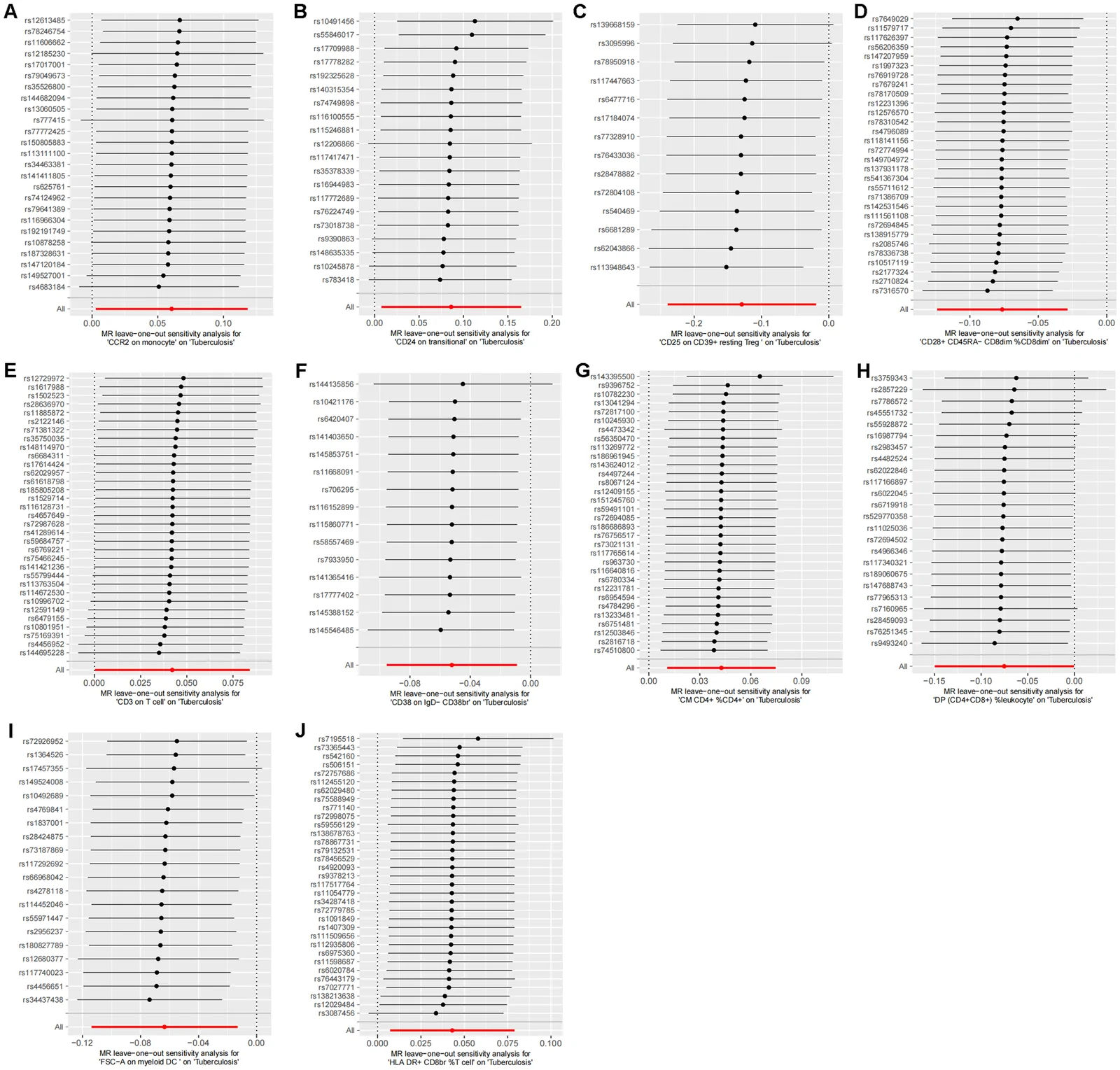
Forest plot for leave-one-out analysis. The position of the red point and the black dots are positioned on the same side of the invalid line, which indicates that removing any of the SNPs will not significantly impact the results. **(A)** The MFI of CCR2 on monocytes; **(B)** The MFI of CD24 on transitional (CD24⁺CD38^high^) B cells; **(C)** The MFI of CD25 on CD39⁺ resting (CD25⁺⁺CD45RA⁺) Treg; **(D)** The percentage of CD28⁺CD45RA⁻CD8^dim^ cells in CD8^dim^ cells; **(E)** The MFI of CD3 on T cells; **(F)** The MFI of CD38 on IgD⁻CD38^bright^ B cells; **(G)** The percentage of CD4⁺ T_CM_ (central memory, CCR7⁺CD45RA⁻) in CD4⁺ T cells; **(H)** The percentage of CD4⁺CD8⁺ DP T cells in leukocytes; **(I)** FSC-A on myeloid DCs; **(J)** The percentage of HLA-DR⁺CD8^bright^ cells in T cells.

Importantly, none of these 10 nominally associated traits met the stringent Bonferroni threshold for robust genetic causality. These weak unadjusted P-value signals may reflect random statistical noise from extensive multiple testing and cannot be interpreted as definitive evidence supporting causal links between genetically determined baseline immune phenotypes and TB susceptibility. Heterogeneity of SNPs was assessed using Q statistic. The results showed that all p-value was > 0.05, which suggested that there was no heterogeneity among IVs (**Table 2**). The MR-Egger intercept test and the MRPRESSO global test, showed absence of horizontal pleiotropy (**Table 3**). Sensitivity analysis using the leave-one-out method confirmed the stability of our findings when systematically removing individual SNPs (**Figure 3**).

**Table 2:** Heterogeneity test of MR.

Exposure	Method	Q	Q_df	Q_pval
CCR2 on monocyte	MR Egger	16.66	23	0.83
	Inverse variance weighted	16.72	24	0.86
CD24 on transitional	MR Egger	14.10	18	0.72
	Inverse variance weighted	14.18	19	0.77
CD25 on CD39+ resting Treg	MR Egger	9.04	12	0.70
	Inverse variance weighted	9.05	13	0.77
CD28+ CD45RA- CD8dim %CD8dim	MR Egger	30.01	28	0.36
	Inverse variance weighted	30.51	29	0.39
CD3 on T cell	MR Egger	17.57	31	0.97
	Inverse variance weighted	17.64	32	0.98
CD38 on IgD- CD38br	MR Egger	5.96	13	0.95
	Inverse variance weighted	6.31	14	0.96
CM CD4+ %CD4+	MR Egger	31.75	30	0.38
	Inverse variance weighted	33.26	31	0.36
DP (CD4+CD8+) %leukocyte	MR Egger	18.66	22	0.67
	Inverse variance weighted	18.66	23	0.72
FSC-A on myeloid DC	MR Egger	28.94	18	0.05
	Inverse variance weighted	29.27	19	0.06
HLA DR+ CD8br %T cell	MR Egger	24.28	31	0.80
	Inverse variance weighted	24.38	32	0.83

**Table 3:** Pleiotropy test of MR.

Exposure	Method	Value	p-value
CCR2 on monocyte	Intercept in MR-Egger regression	-0.003	0.81
	RSSobs in MR-PRESSO global test	17.41	0.87
CD24 on transitional	Intercept in MR-Egger regression	-0.006	0.78
	RSSobs in MR-PRESSO global test	16.25	0.73
CD25 on CD39+ resting Treg	Intercept in MR-Egger regression	0.003	0.89
	RSSobs in MR-PRESSO global test	10.13	0.80
CD28+ CD45RA- CD8dim %CD8dim	Intercept in MR-Egger regression	0.006	0.50
	RSSobs in MR-PRESSO global test	32.09	0.41
CD3 on T cell	Intercept in MR-Egger regression	-0.002	0.80
	RSSobs in MR-PRESSO global test	18.86	0.98
CD38 on IgD- CD38br	Intercept in MR-Egger regression	-0.006	0.56
	RSSobs in MR-PRESSO global test	6.76	0.98
CM CD4+ %CD4+	Intercept in MR-Egger regression	0.008	0.24
	RSSobs in MR-PRESSO global test	37.25	0.39
DP (CD4+CD8+) %leukocyte	Intercept in MR-Egger regression	0.000	0.99
	RSSobs in MR-PRESSO global test	19.56	0.77
FSC-A on myeloid DC	Intercept in MR-Egger regression	0.005	0.65
	RSSobs in MR-PRESSO global test	31.31	0.10
HLA DR+ CD8br %T cell	Intercept in MR-Egger regression	-0.003	0.76
	RSSobs in MR-PRESSO global test	26.61	0.80

Interpretation of these nominal unadjusted genetic associations must account for the European ancestry origin of all GWAS summary data. Causal signals identified in European populations cannot be uncritically extrapolated to East Asian TB cohorts without independent replication in local genetic datasets.

We prioritized CD4^+^CD8^+^ DP T cells from these hits for comprehensive clinical and functional analysis out of combined academic and practical considerations. Our group has long investigated T cell-mediated anti-Mtb immunity; three of the ten candidate markers are well-described conventional single-positive T cell parameters with limited novelty, whereas peripheral DP T cells remain an understudied unconventional T subset in East Asian active TB cohorts (Simonson et al., 2025; Zou et al., 2022). Standardized six-color flow cytometry data for DP subsets were uniformly archived for all participants, while the extended multi-color antibody panels required to detect the other nine immune signatures were not routinely implemented, leaving no complete matched datasets to support parallel comparative analysis.

Crucially, our clinical immunological observations address infection-triggered immune perturbations among East Asian TB patients, a research question distinct from the germline genetic causal inference generated by European GWAS datasets. While the nominal MR associations failed Bonferroni correction and cannot support causal evidence for innate genetic TB predisposition, this large-scale unbiased screening still provided an exploratory candidate pool to guide our subsequent T cell-focused validation.

### 3.2 Peripheral lymphocyte alterations and diagnostic value in active TB

**Figure 4:**
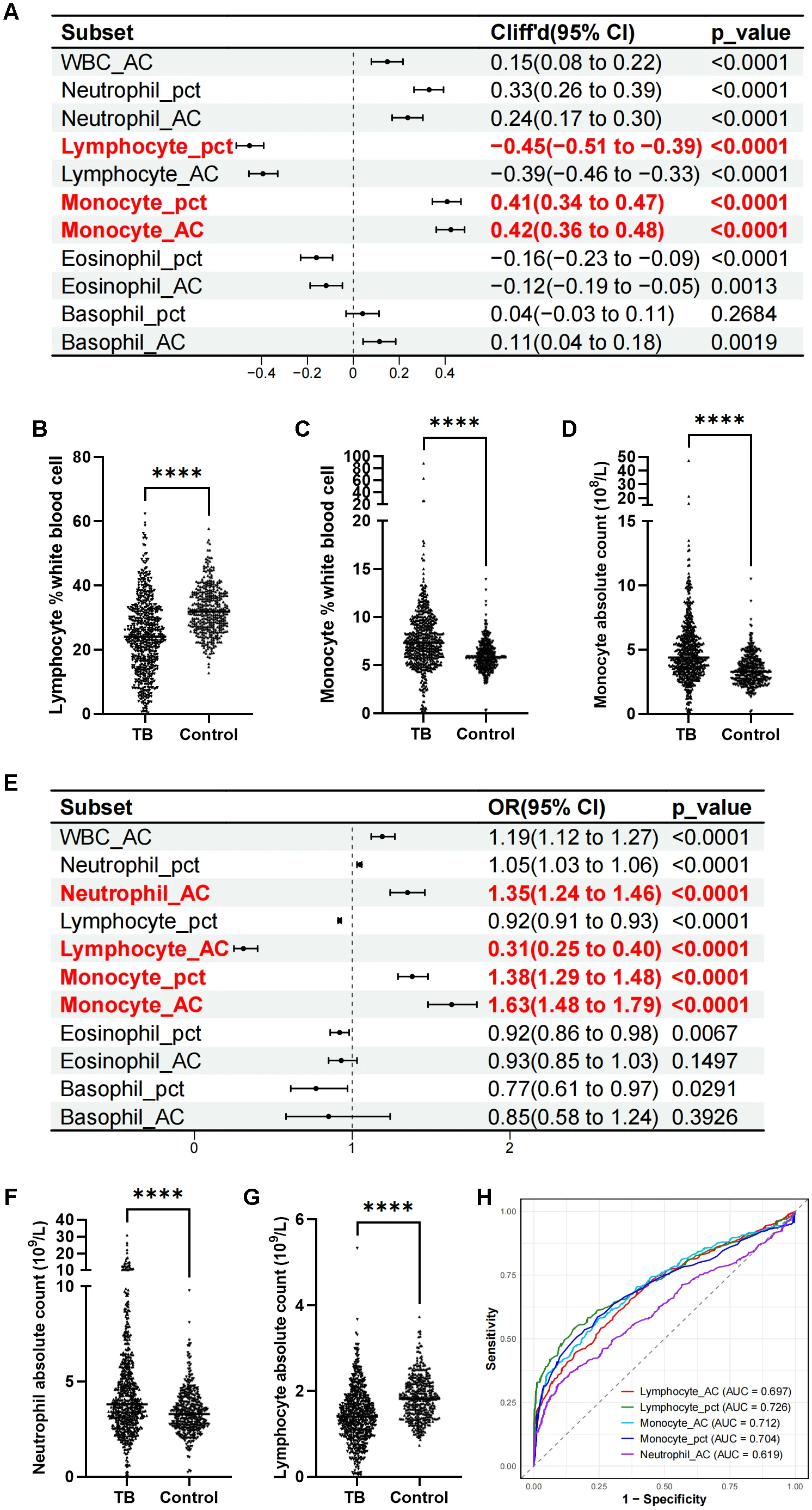
Percentage of lymphocytes in leukocytes was decreased in patients with TB. **(A)** Forest plot of the difference effect size Cliff’δ of the subsets of peripheral leukocytes. **(B)** Percentage of lymphocytes in the leukocytes in patients with TB and healthy controls. **(C)** Percentage of monocytes in the leukocytes in patients with TB and healthy controls. **(D)** Absolute count of monocytes in the peripheral blood in patients with TB and healthy controls. **(E)** Forest plot of the univariate logistic regression results of the subsets of peripheral leukocytes. **(F)** Absolute count of neutrophils in patients with TB and healthy controls. **(G)** Absolute count of lymphocytes in the peripheral blood in patients with TB and healthy controls. **(H)** ROC curves of parameters in leukocytes for distinguishing active TB from healthy controls. ****P<0.0001.

To explore the peripheral blood immune landscape of active TB patients, the absolute counts and proportions of granulocytes (eosinophils and basophiles), monocytes and lymphocytes were compared between active TB patients and healthy controls. The results showed that: Monocytes absolute counts were significantly elevated in TB patients (Cliff’s δ = 0.42, OR = 1.63, p < 0.0001), accompanied by prominent expansion in neutrophil absolute counts (Cliff’s δ = 0.24, OR = 1.35, p < 0.0001). Conversely, percentage of lymphocyte in leukocytes was significantly reduced in TB cases (Cliff’s δ = −0.45, OR = 0.31, p < 0.0001) (**Figure 4**). ROC analysis further validated the moderate auxiliary diagnostic performance of these altered leukocyte markers for discriminating active TB from healthy individuals (**Figure 4H**): percentage of lymphocyte in leukocytes exhibited the highest predictive efficacy with an AUC of 0.726, followed by monocyte absolute count (AUC = 0.712), monocyte percentage (AUC = 0.704).

**Figure 5:**
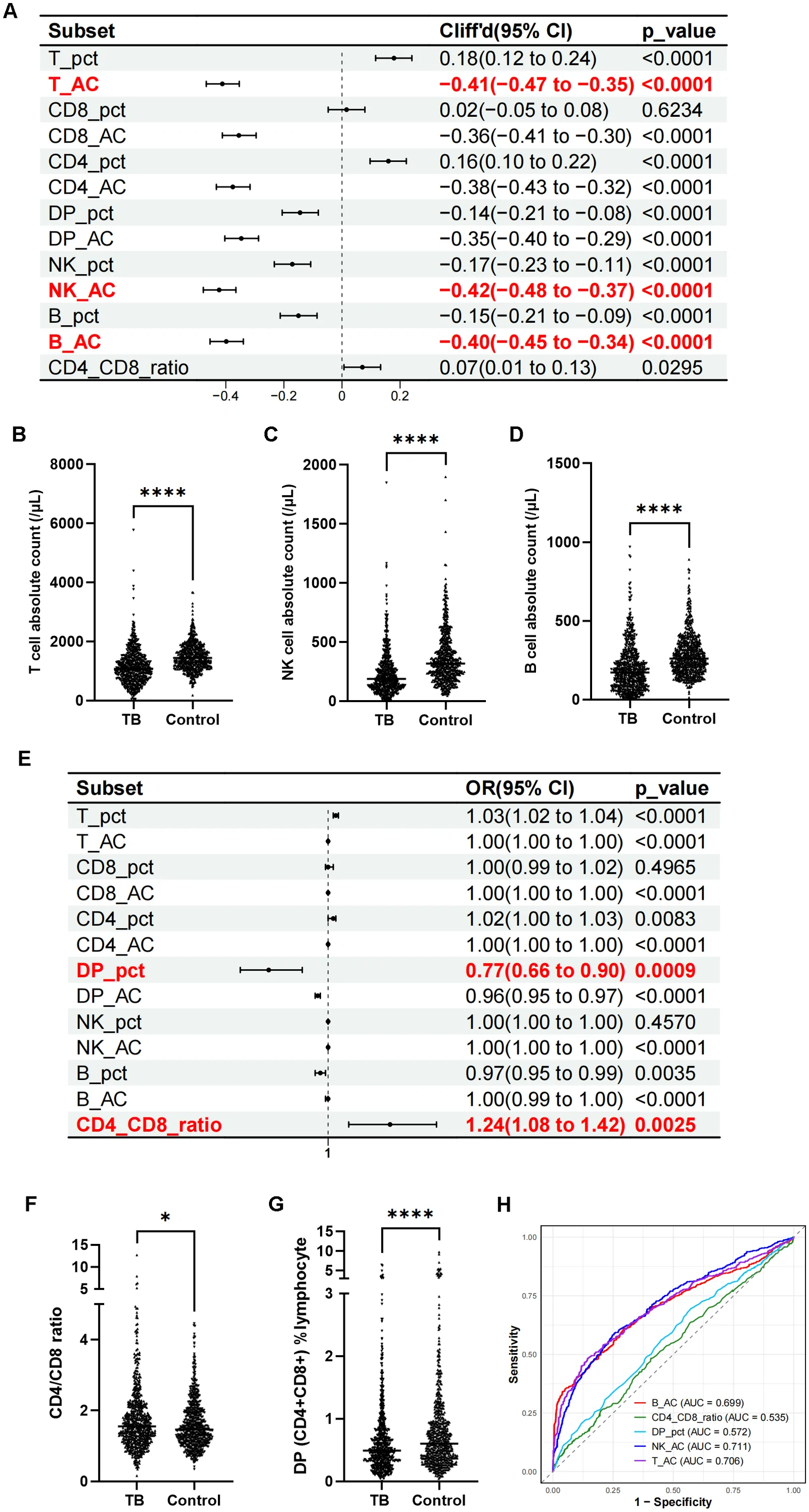
CD4^+^CD8^+^ DP T cells were significantly lower in patients with active TB. **(A)** Forest plot of the difference effect size of the subsets in lymphocyte. **(B–D)** Absolute counts of T cells, NK cells and B cells in the peripheral blood in patients with TB and healthy controls. **(E)** Forest plot of the univariate logistic regression results of the subsets in lymphocyte. **(F)** Ratio of CD4^+^ T cells to CD8^+^ T cells in patients with TB and healthy controls. **(G)** Percentage of DP T cells in lymphocytes in patients with TB and healthy controls. **(H)** ROC curves of parameters in lymphocytes for distinguishing active TB from healthy controls. *P<0.05, ****P<0.0001.

Further analysis of lymphocyte subsets showed that absolute count of T cells (Cliff’s δ= -0.41, p<0.0001), B cells (Cliff’s δ= -0.40, p<0.0001) and NK (Cliff’s δ= -0.42, p<0.0001) cells were significantly lower in patients with active TB (**Figures 5A–D**). Univariate logistic regression analysis revealed that ratio of CD4/CD8 T cells was significantly higher in the patients with TB, whereas percentage of DP T cells in lymphocytes was significantly lower in the patients with TB than healthy controls (**Figures 5E–G**). ROC curve showed that the absolute count of T cells (AUC = 0.706), B cells (AUC = 0.699) and NK cells (AUC = 0.711) could serve as potential diagnostic biomarker for active TB (**Figure 5H**).

Collectively, these pan-leukocyte analyses define a distinct peripheral immune hallmark of active TB: prominent expansion of neutrophils and monocytes alongside broad lymphocyte depletion. This panel of perturbed leukocyte signatures jointly functions as reliable auxiliary biomarkers to distinguish patients with active TB from healthy controls.

### 3.3 Percentage of DP (CD4^+^CD8^+^) T cells in leukocytes was associated with disease severity and prognosis of TB

Based on the global peripheral lymphocyte immune landscape characterized above, we further focused on DP T cells to systematically explore their correlations with multiple TB clinical phenotypes covering disease severity and treatment response. This unconventional T subset was prioritized as a core research target from MR screening owing to its insufficiently investigated biological roles in East Asian active TB cohorts.

**Figure 6:**
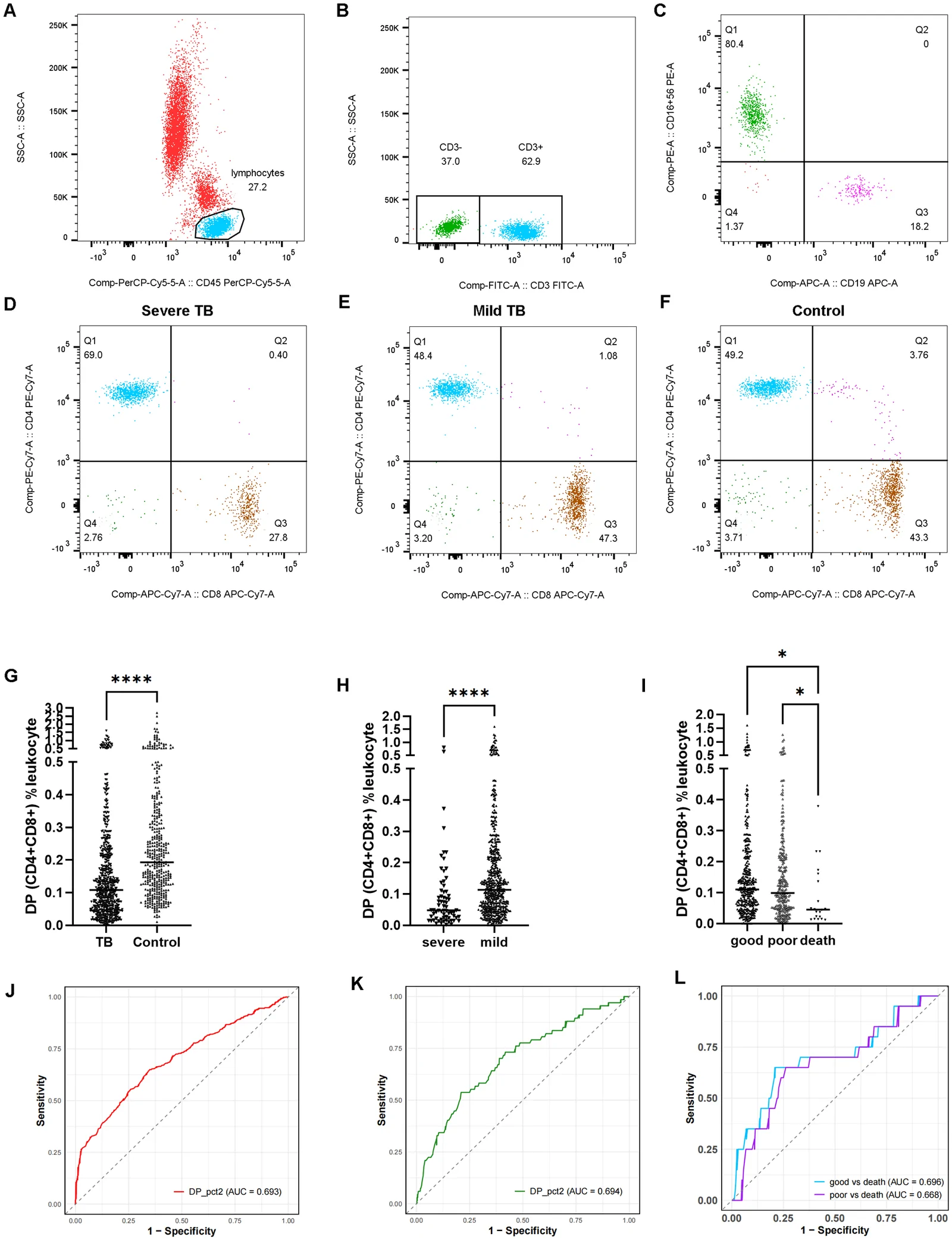
The percentage of DP T cells in leukocytes were significantly lower in patients with severe TB. **(A)** Gate strategy of lymphocytes. Leukocytes (CD45^+^ cells) were divided into lymphocytes, monocytes and neutrophils on the basis of morphological parameters side scatter (SSC)-A. **(B)** Lymphocytes were divided into CD3^-^ and CD3^+^ cells. **(C)** The CD3^-^ cells then split into NK cells (CD19^-^ CD56^+^) and B cells (CD19^+^CD56^-^). The CD3^+^ cells corresponding to T cells, and then T lymphocytes were split into four subsets based on the expression of the CD4 and CD8 markers: CD4^−^CD8^−^ (DN), CD4^+^CD8^−^ (CD4 SP), CD4^−^CD8^+^ (CD8 SP) and CD4^+^CD8^+^ (DP). Representative flow cytometric plots showing the percentage of DP T cells in patients with severe TB **(D)**, mild TB **(E)** and healthy controls **(F)**. **(G)** Percentage of DP T cells in leukocytes in patients with TB and healthy controls. **(H)** Percentage of DP T cells in leukocytes in patients with severe TB and mild TB. severe TB: requiring invasive mechanical ventilation or comorbid with one or multiple organ failures; mild TB: No need for invasive mechanical ventilation and no organ failure. **(I)** Percentage of DP T cells in leukocytes in patients with different treatment efficacy. Good: the pathogen detection turned negative or the lesion shows absorption within one month of treatment; poor: the pathogen detection did not turn negative, or the lesion showed no absorption and even increased after one month of treatment; death: the patient died in the hospital within one month after sampling. **(J)** ROC curves of DP T cells in leukocytes for distinguishing active TB from healthy controls. **(K)** ROC curves of DP T cells in leukocytes for differentiating severe TB from mild TB. **(L)** ROC curves of DP T cells in leukocytes for distinguishing patients died within one month from patients with good treatment efficacy (blue) or poor treatment efficacy (purple). *P<0.05, ****P<0.0001.

Flow cytometric analysis of the lymphocyte subsets showed that the percentage of DP T cells in leukocytes was significantly lower in the patients with TB than healthy controls (**Figure 6G**). We further stratified participants by IGRA and infection status into IGRA-positive activeTB (TB_IGRA+), IGRA-negative active TB (TB_IGRA–), asymptomatic IGRA+ latent TB infection (LTBI), and IGRA-negative healthy controls for intergroup DP T comparison (**Supplementary Figure 1**). Numerically, LTBI and TB_IGRA+ groups presented slightly higher median DP T frequencies, but all pairwise comparisons showed no statistical significance (p > 0.05, ns), which may relate to incomplete IGRA testing and unbalanced subgroup sample sizes.

Further subgroup analysis revealed that percentage of DP T cells in leukocytes was notably lower in the patients with severe TB and in dividual who died within one month after admission, compared with those with mild disease (**Figures 6H, I**). ROC curve analysis indicated that DP T cells percentage in leukocytes exhibited moderate diagnostic efficacy for distinguishing active TB from healthy controls (AUC = 0.693). Additionally, it showed potential prognostic value for stratifying disease severity (AUC = 0.694) and evaluating treatment response (AUC = 0.696) (**Figures 6J–L**).

### 3.4 DP T cells were positively correlated with IFN-γ production

**Figure 7:**
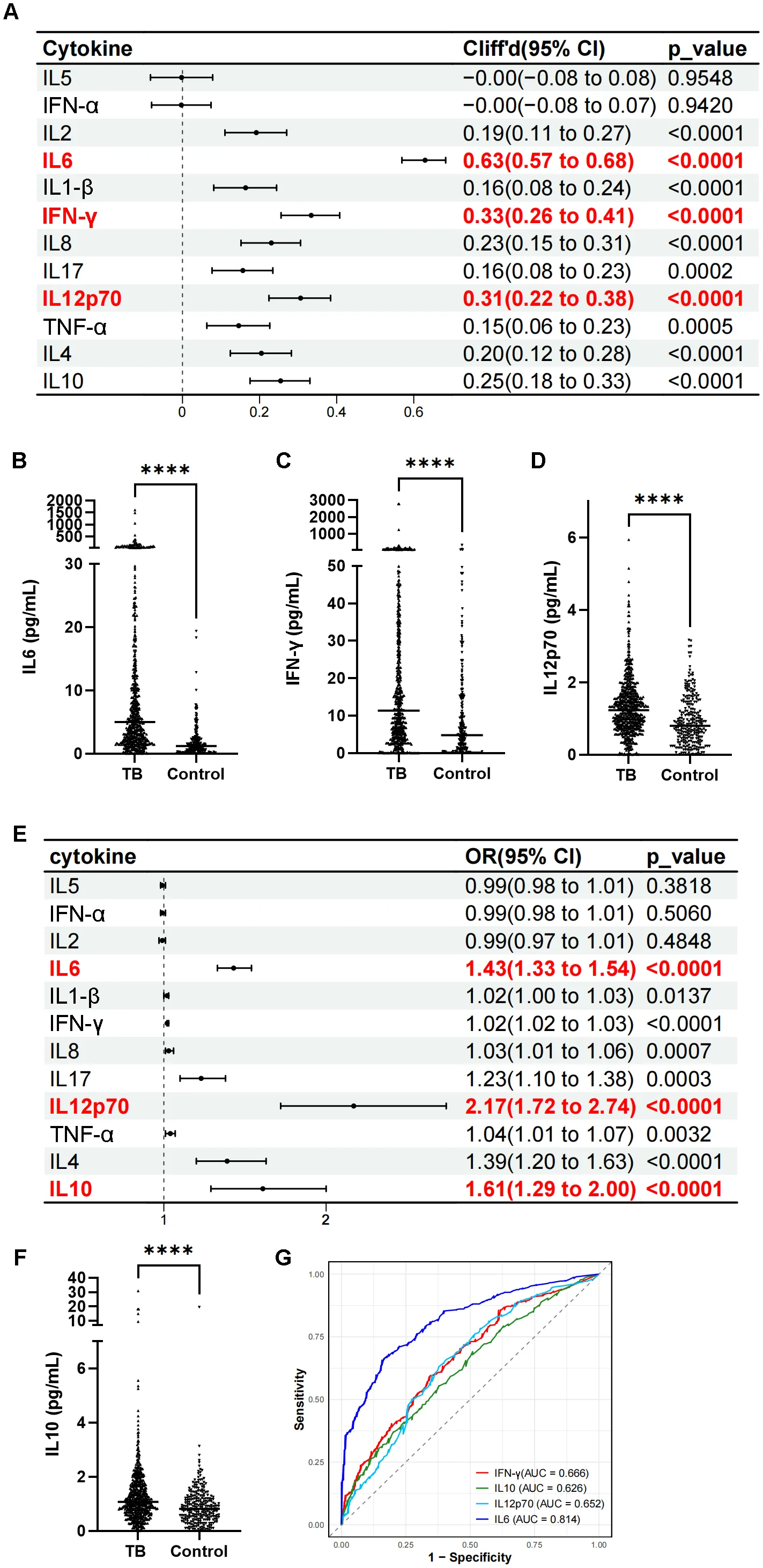
Plasma cytokine profiles in patients with active tuberculosis (TB) and healthy controls. **(A)** Forest plot showing effect sizes (Cliff’s δ) for differences in plasma cytokine levels. **(B–D)** Comparison of plasma IL-6, IFN-γ, and IL-12p70 concentrations between active TB patients and healthy controls. **(E)** Forest plot of univariate logistic regression analyses for plasma cytokine levels. **(F)** Comparison of plasma IL-10 concentrations between active TB patients and healthy controls. **(G)** ROC curves of IL-6, IFN-γ, IL-12p70, and IL-10 for discriminating active TB from healthy controls. ****p < 0.0001.

Concentration of plasma cytokines in patients with active TB and healthy controls were quantified by multiplexed flow cytometric immunofluorescence assay. The concentration of IL-6 (Cliff’s δ= 0.63, p<0.0001), IFN-γ (Cliff’s δ= 0.33, p<0.0001), IL-12p70 (Cliff’s δ= 0.31, p<0.0001) in plasma was significantly higher in the patients with TB than healthy controls (**Figures 7A–D**). Univariate logistic regression showed that IL-6 (OR (95% CI) =1.43 (1.33 to 1.54), p<0.0001), IL-12p70 (OR (95% CI) =2.18 (1.73 to 2.75), p<0.0001) and IL-10 (OR (95% CI) =1.62 (1.30 to 2.01), p<0.0001) were risk factors for active TB (**Figures 7E, F**). ROC analysis yielded AUC values of 0.814 for IL-6, 0.666 for IFN-γ, and 0.652 for IL-12p70, indicating their potential diagnostic performance for distinguishing active TB from healthy individuals (**Figure 7G**).

**Figure 8:**
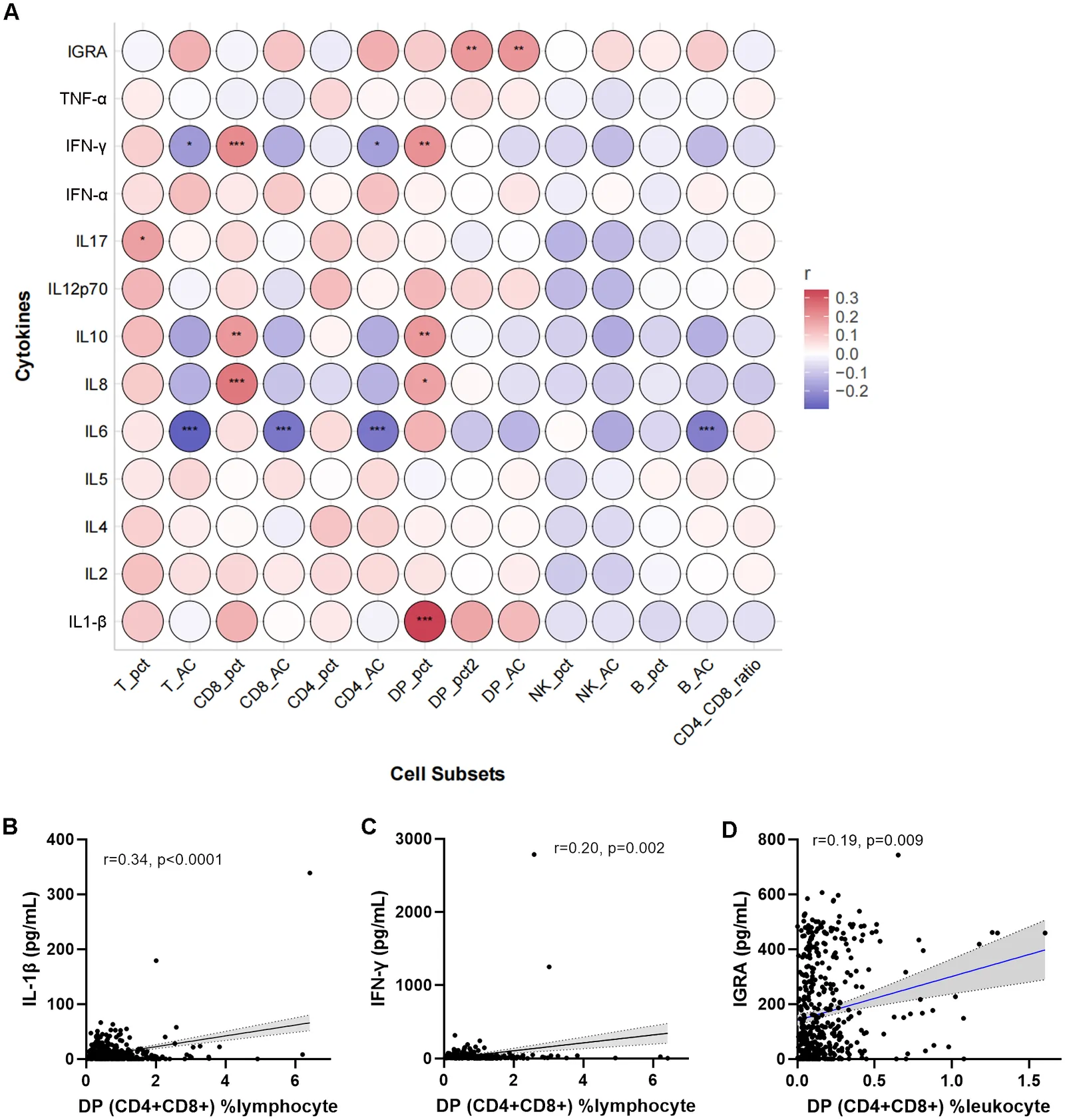
The percentage of DP T cells in leukocytes was positively correlated with Mtb-specific IFN-γ production. **(A)** Pearson correlation analysis between lymphocyte subsets and plasma cytokines. The color gradient represents the Pearson correlation coefficient, and asterisks denote statistical significance. *p < 0.05; **p < 0.01; ***p < 0.001. **(B, C)** Positive correlations of DP T cell percentage in lymphocytes with plasma IL-1β and IFN-γ concentrations. **(D)** Positive correlation between DP T cell percentage in leukocytes and Mtb-specific antigen-induced IFN-γ production.

To explore the correlation between lymphocyte subsets and plasma cytokines in patients with active TB, Pearson correlation analysis was conducted using the R “correlation” package, and a correlation heatmap was visualized via the R “ggplot2” package (**Figure 8A**). Absolute counts of T cells (r = −0.20, p < 0.001), CD8 single-positive (SP) T cells (r = −0.16, p < 0.001), CD4 SP T cells (r = −0.19, p < 0.001), and B cells (r = −0.16, p < 0.001) were negatively correlated with IL-6 levels. The percentage of DP T cells among lymphocytes showed significant positive correlations with IL-1β (r = 0.34, p < 0.001) (**Figure 8B**), IFN-γ (r = 0.20, p = 0.002) (**Figure 8C**), and IL-10 (r = 0.19, p = 0.009). In particular, both the absolute count (r = 0.20, p = 0.005) and percentage of DP T cells in leukocytes (r = 0.19, p = 0.009) were positively associated with Mtb antigen-specific IFN-γ production (**Figure 8D**).

**Figure 9:**
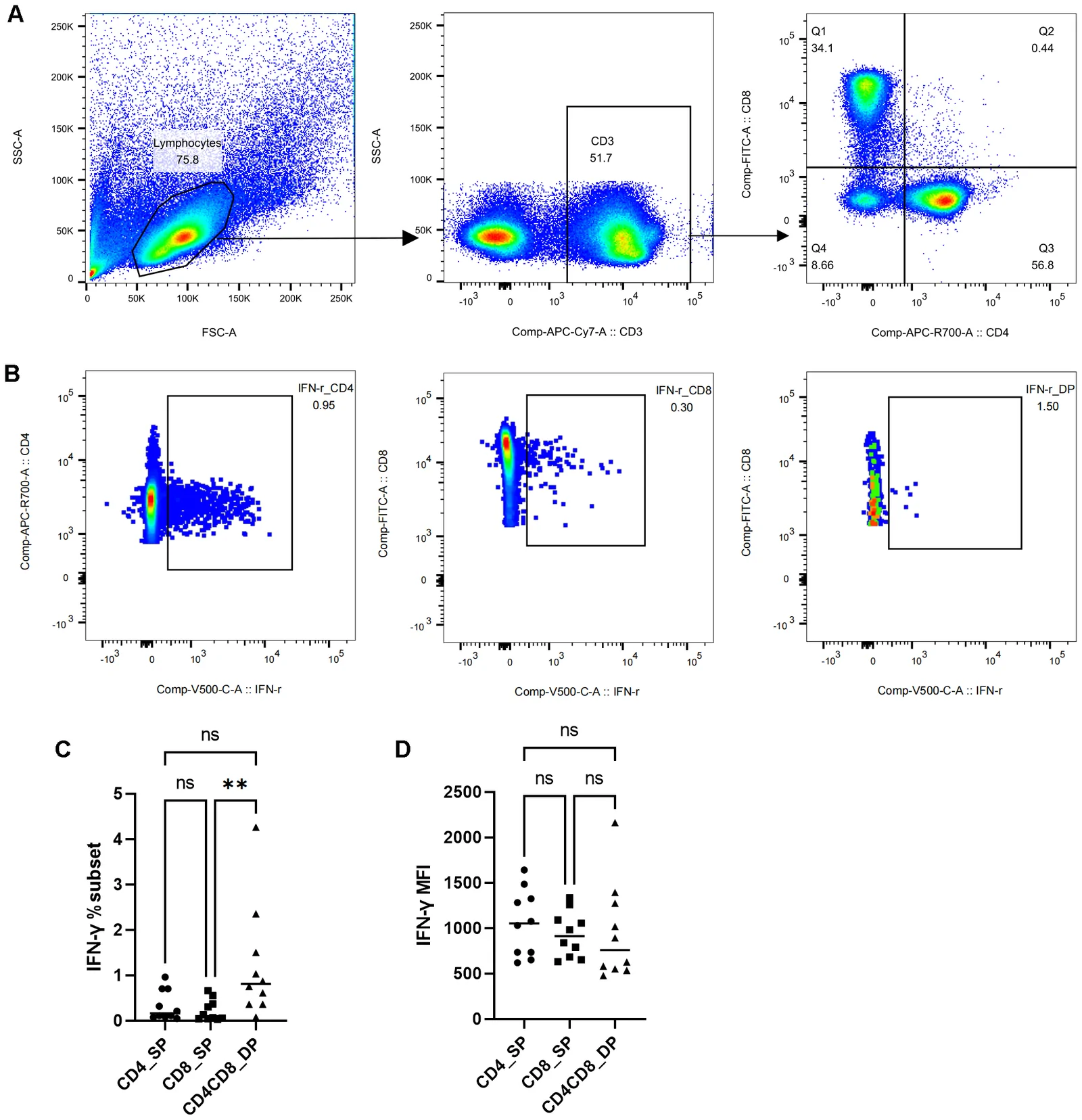
Flow cytometric analysis of IFN-γ production in T cells subsets. **(A)** Gating strategy for the T cells. PBMCs were first gated for lymphocytes based on FSC-A and SSC-A, reflecting cell size and intracellular complexity, respectively. Lymphocytes were further defined as CD3^+^ T cells, which were subdivided into four subsets according to the expression of the CD4 and CD8: CD4^−^CD8^−^ (DN), CD4^+^CD8^−^ (CD4 SP), CD4^−^CD8^+^ (CD8 SP) and CD4^+^CD8^+^ (DP). **(B)** Representative flow cytometric plots showing expression of IFN-γ in CD4 SP (left), CD8 SP (middle) and DP (left) T cells. **(C)** Frequencies of IFN-γ^+^ cells in CD4 SP, CD8 SP and DP T cells. **(D)** Mean fluorescence intensity of IFN-γ^+^ cells in CD4 SP, CD8 SP and DP T cells. ns, no significantly difference; **p<0.01.

To clarify the role of DP T cells in IFN-γ production, T cells were stimulated with CD3/CD28 antibodies. Flow cytometric analysis revealed that the percentage of IFN-γ-producing DP T cells was significantly higher than that of CD8 SP T cells. No statistically significant difference was observed between the percentage of IFN-γ^+^ DP T cells and CD4 SP T cells, despite numerically higher IFN-γ frequencies in the DP subset (**Figures 9B, C**). The mean fluorescence intensity (MFI) of IFN-γ in the three subsets of T cells was similar (**Figure 9D**). These findings suggested that DP T cells possess stronger IFN-γ secretion capacity relative to CD8^+^ SP T cells, which may contribute to a more balanced antimycobacterial immune profile during TB infection.

Notably, cross-sectional correlation analysis in this retrospective cohort cannot distinguish the bidirectional regulatory direction between DP T cell abundance and IFN-γ levels. The European-population MR analysis only evaluates germline baseline immune predisposition and cannot recapitulate dynamic post-infection immune feedback after Mtb infection, so the causal relationship between DP T cells and IFN-γ secretion remains unclarified in the present study.

### 3.5 Enhancing prognostic accuracy for TB using random forest models incorporating 7 peripheral blood features

To further integrate the screened immune signatures and build predictive models for auxiliary clinical evaluation of TB severity and prognosis, we further established random forest (RF) models to improve the prognostic evaluation of TB based on seven peripheral blood biomarkers with AUC > 0.65, including the percentages of DP T cells, lymphocytes and monocyte in leukocytes, plasma IL-6 concentration, and absolute counts of lymphocytes, NK cells, and T cells. Model hyperparameters were optimized via repeated cross-validation (repeated cv) with 5-fold cross-validation to maintain data robustness and reduce overfitting.

**Figure 10:**
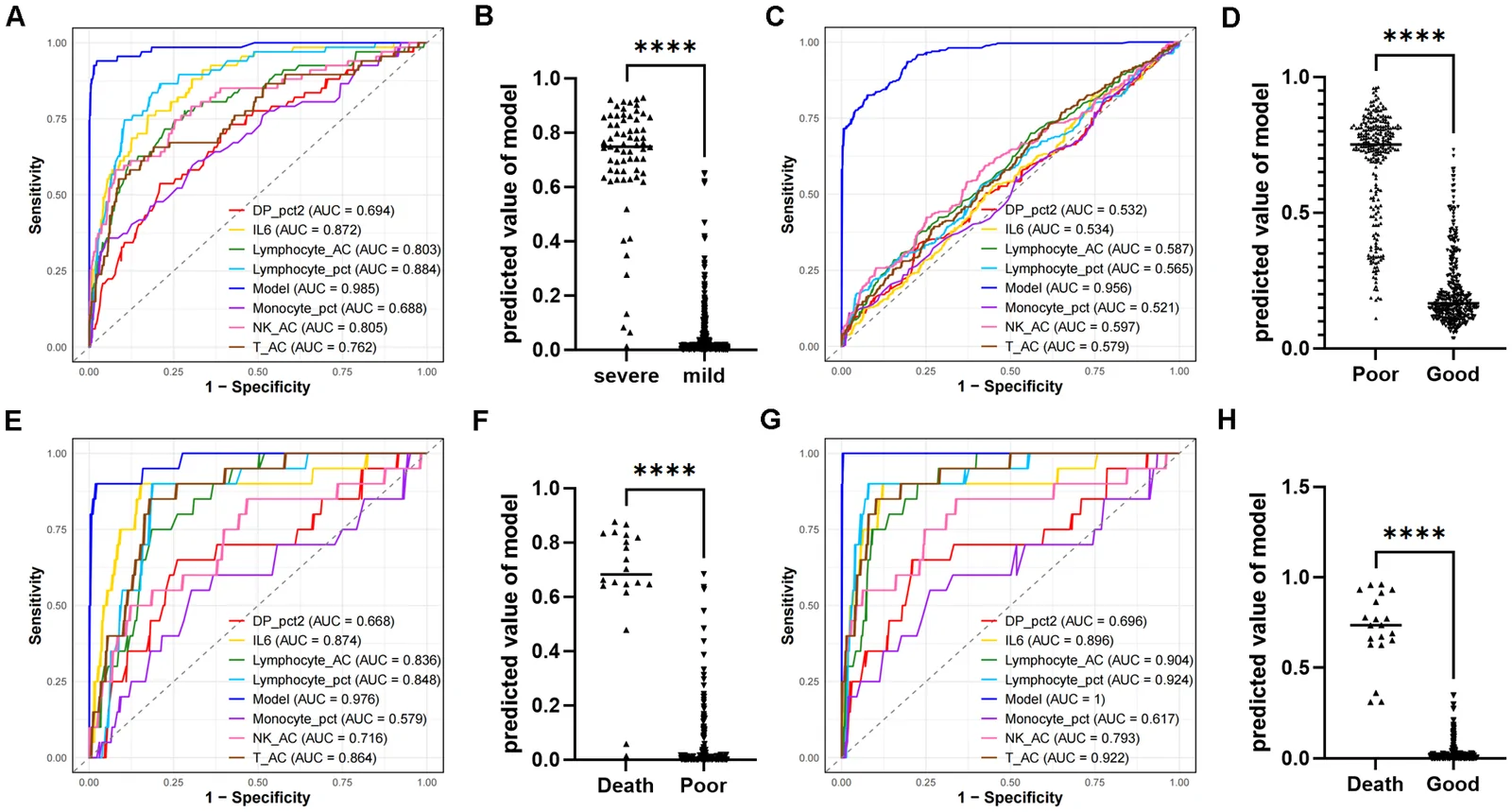
Performance of the random forest (RF) models for prognosis and therapy effect in active TB. **(A)** ROC curves of the seven parameters (percentages of DP T cells, lymphocytes and monocytes in leukocytes, the absolute number of lymphocytes, NK cells and B cells; the plasma IL-6 concentration), as well as the integrated RF model combining all seven indicators, for distinguishing severe TB from mild TB. Severe TB was defined as patients requiring invasive mechanical ventilation or comorbid with one or multiple organ failures; mild TB was defined as no need for invasive mechanical ventilation and absence organ failure. **(B)** Distribution of the RF model scores for individual patients with severe TB and mild TB. **(C, E, F)** ROC curves of the seven single parameters and the combined RF model for distinguishing patients with different treatment outcome. Poor: pathogen detection remained positive or pulmonary lesions showed no absorption or even enlargement after one month of anti-TB treatment. Good: pathogen conversion to negative or obvious lesion absorption within one month of treatment. Death: in-hospital death within one month after sample collection. **(D, F, H)** Distribution of the RF model scores for individual samples from different treatment outcomes. ****P<0.0001.

To discriminate severe TB from mild TB, the RF model adopted a decision threshold of 0.275. At this cutoff, the model yielded an AUC of 0.985 (95% CI: 0.970-1.000), a sensitivity of 94.03% (95% CI: 85.63%-97.65%), and a specificity of 97.73% (95% CI: 96.15%-98.67%) (**Figures 10A, B**).

For distinguishing TB patients with good from poor treatment efficacy, RF model showed an AUC of 0.956 (95% CI, 0.942–0.970), sensitivity of 82.58% (95% CI, 77.54%–86.68%), and specificity of 92.39% (95% CI, 89.16%–94.72%) at a cutoff value of 0.449 (**Figures 10C, D**).

As shown in **Figures 10E, F**, at a cutoff value of 0.295, the RF model achieved an AUC of 0.976 (95% CI, 0.946–1.000), sensitivity of 90.00% (95% CI, 69.90%–98.22%) and specificity of 98.11% (95% CI, 95.64%–99.19%) for differentiating TB patients with poor treatment efficacy from death. With a cutoff value of 0.304, the model attained an AUC of 1.000 (95% CI, 0.999–1.000), sensitivity of 100% (95% CI, 83.89%–100%) and specificity of 99.72% (95% CI, 98.42%–99.99%) for differentiating TB patients with good treatment efficacy from death (**Figures 10G, H**).

Notably, the integrated RF models exhibited obviously better predictive performance than using DP T cells alone (**Figure 10**).

## 4 Discussion

In the present study, we used a comprehensive dataset comprising 731 immune cell traits (Orru et al., 2020), including 118 absolute cell counts, 192 relative cell proportions, 389 median fluorescence intensities (MFIs) of surface markers, and 32 morphological parameters, to explore the causal relationships between these immune phenotypes and TB via Mendelian randomization. At the unadjusted nominal threshold of raw IVW p<0.05, ten immune cell features displayed suggestive associations with TB susceptibility; however, all these nominal signals failed to meet the stringent Bonferroni cutoff for robust genetic causality. Among these nominally correlated traits, we prioritized CD4^+^CD8^+^ double-positive (DP) T cells for independent clinical validation based on unique biological novelty and complete unified flow cytometry records available in our retrospective cohort. Clinical validation demonstrated that the percentage of DP T cells in leukocytes was markedly reduced in active TB patients and inversely correlated with TB disease severity and treatment response (**Figure 6**). Multiple immune alterations triggered by Mtb collectively reduce circulating DP T cell frequencies in patients with active TB. Mtb infection drives pulmonary granuloma formation and induces abundant chemokine ligands within lesions; DP T cells highly express CXCR3 and CCR5 receptors, enabling robust recruitment from peripheral blood to local inflammatory granuloma niches (Zou et al., 2022). In addition, persistent stimulation by intracellular mycobacterial antigens drives widespread T cell apoptosis and impairs peripheral lymphoid differentiation programs, further limiting the replenishment of circulating DP T pools (Li et al., 2023a, b). Such tissue sequestration mechanism is rarely observed in systemic viral infections, which explains the opposite DP T dynamics between TB and viral diseases.

It is well established that CD4^+^CD8^+^ DP and CD4^-^CD8^-^ double-negative T cells are conventionally regarded as thymus-resident cells. However, mature peripheral CD4^+^CD8^+^ DP T cells have been detected in healthy individuals (Blue et al., 1985) and distinct diseases (Hess et al., 2023). Systemic viral infections induce expansion of circulating DP T cells: COVID-19 patients present elevated DP frequency accompanied by prolonged fever (Zahran et al., 2021); dengue virus infection increases peripheral DP T proportion, which acts as an independent risk marker for plasma leakage (Yu et al., 2022); Hantaan virus infection also drives CD8-lineage DP T proliferation with enhanced cytotoxic function (Zhang et al., 2022); similarly, HIV monoinfection and HIV-TB co-infection feature expanded CD4^low^CD8^high^ DP subsets (Zou et al., 2022). Autoimmune disorders such as primary Sjögren’s syndrome also show higher peripheral DP T cells levels correlated with milder disease activity (Wang et al., 2021). In malignancies, tumor border-enriched PD-1^high^ DP T cells in hepatocellular carcinoma exert anti-tumor effector functions and predict favorable survival (Zheng et al., 2020). In the current study, we found that the percentage of peripheral DP T cells in leukocytes was markedly decreased in patients with active TB and inversely correlated with disease severity. Such divergent DP T dynamics might stem from fundamental differences in the immune microenvironment and pathogen-specific immune evasion mechanisms between intracellular Mtb and acute or chronic viruses. Viruses replicate widely in the bloodstream and mucosal compartments, triggering systemic inflammatory cascades that sustain peripheral DP T proliferation and preserve their cytotoxic capacity (Zhang et al., 2022; Frahm et al., 2012). To evade host immunity, viruses primarily suppress antigen presentation and disrupt intracellular interferon responses, and they do not establish persistent, walled-off local lesions. As a result, DP T cells accumulate in peripheral circulation to eliminate systemically disseminated virions. In contrast, Mtb drives encapsulated pulmonary granuloma formation as a core immune evasion strategy. Granuloma-derived chemokines recruit CXCR3/CCR5-positive DP T cells from circulation to lung lesions, lowering peripheral DP T counts (Zou et al., 2022). Chronic antigen stimulation inside granulomas also triggers T cell apoptosis and impairs lymphoid progenitor development, limiting peripheral DP T renewal (Li et al., 2023b). Together, tissue trapping and increased apoptosis probably account for depleted circulating DP T cells during active TB.

Accumulating evidence has demonstrated that DP T cells from HIV-infected individuals exhibit enhanced proliferative capacity (Frahm et al., 2012; Weiss et al., 1998), while DP T cells in hemorrhagic fever patients express higher levels of Granzyme B and CD107a and display a more diverse TCR repertoire (Zhang et al., 2022). DP T cells can secrete multiple cytokines including IFN-γ, TNF-α, IL-4, and IL-21 in rheumatoid arthritis and HIV infection (Weiss et al., 1998; Quandt et al., 2014), indicating that DP T cells are functionally competent and may contribute to host defense against infectious pathogens. In our cohort, the percentage of DP T cells in lymphocytes was positively correlated with plasma levels of IL-1β, IFN-γ, and IL-8 (**Figure 8G**). Furthermore, Mtb antigen-specific IFN-γ production was positively associated with the percentage of DP T cell among leukocytes based on IGRA results (**Figure 8J**). *In vitro* functional experiments further showed that DP T cells contained a significantly larger fraction of IFN-γ-producing cells than CD8^+^ single-positive (SP) T cells; while DP T cells exhibited numerically higher IFN-γ^+^ than CD4^+^ SP T cells, this contrast lacked statistical significance (**Figure 9**). Numerous studies have delineated the multifaceted downstream regulatory networks of IFN-γ among distinct immune cell subsets. Mechanistically, IFN-γ activates the STAT1-IRF1 signaling axis in macrophages, which drives the generation of ROS, initiates autophagic pathways and upregulates a panel of antimicrobial effectors to eliminate intracellular mycobacteria (Imran et al., 2025; Rosain et al., 2023). In tuberculous granulomas, IFN-γ orchestrates extensive intercellular communication across multiple immune lineages. On one hand, IFN-γ limits the immunosuppressive function of Tregs. Without such restraint, Mtb utilizes active Tregs to suppress macrophage bactericidal activity (Cheng et al., 2025). On the other hand, IFN-γ promotes the maturation of cDC1 and their secretion of IL-12, consequently amplifying protective Th1 immune responses (Imran et al., 2025). However, monocyte-derived myeloid cells possess an intrinsic defect in responding to IFN-γ. Such cell populations act as long-term bacterial reservoirs and ultimately impair the overall host defense against mycobacterial infection (Zheng et al., 2026). Given the well-documented protective role of IFN-γ in TB immunity, our collective clinical and *in vitro* data suggested that DP T cells may shape balance anti-TB immune profiles through promoting IFN-γ secretion. Consistently, a previous study in macaques demonstrated that depletion of unconventional CD8α-expressing lymphocytes, including NK cells, innate T cells, and CD4^+^CD8α^+^ DP T cells, weakened host immune control over Mtb infection (Simonson et al., 2025).

Compared with using the percentage of DP T cell alone, the random forest (RF) model integrating DP T cells and six additional peripheral blood immune features substantially improved the sensitivity and specificity for discriminating severe from mild active TB (**Figure 10**), which may facilitate timely clinical stratification and individualized intervention. Although our RF model shows promising potential as an accurate machine-learning tool for TB prognostic prediction, its performance relies heavily on the quality and consistency of input data. Further external validation across multiple clinical centers is therefore required to verify its generalizability and diagnostic robustness.

From a translational perspective, our DP T cell-related biomarkers show promising clinical value. Firstly, we established a cost-effective six-color flow cytometry protocol to quantify DP T cells, which can be readily deployed at primary medical centers for mass TB screening. Secondly, peripheral DP T frequency correlates with disease severity and therapeutic outcomes of active TB, and may serve as a supplementary marker to identify latent TB carriers in future work, pending validation in independent prospective cohort. Thirdly, immunotherapies that expand DP T populations and enhance their IFN-γ secretion are promising strategies for treating drug-resistant and refractory TB. Notably, the random forest model constructed in this study integrates both routine hematological indicators and flow cytometry-detected lymphocyte subsets. Further optimization could streamline the model to generate a concise risk scoring system suitable for rapid bedside clinical evaluation.

Our MR analysis was based on a large database involving 477,386 individuals and 24,189,689 SNPs, providing sufficient statistical power and population representativeness. Multiple statistical strategies were applied to minimize horizontal pleiotropy and confounding biases, strengthening the reliability of our causal inference (**Tables 2**, **3**). Several limitations of this study should be acknowledged. First, all GWAS summary statistics were exclusively obtained from European ancestry cohorts, which restricts the cross-population generalizability of our causal inference and means these conclusions cannot be directly extrapolated to East Asian TB populations. Second, the potential immunomodulatory effects of DP T cells during TB have only been assessed through *in vitro* functional assays; *in vivo* animal models are still required to confirm how these cells regulate TB disease progression. Third, this single-center cohort lacks multi-center external validation; long-term follow-up data for predicting drug resistance and TB relapse, as well as subgroup analyses stratified by drug susceptibility and anti-TB treatment history, are absent. Fourth, while the MR analysis yielded suggestive causal associations, residual reverse causality between DP T cell frequency and IFN-γ production cannot be completely excluded in the absence of interventional experimental verification. Fifth, blood samples were collected within 3 days after inpatient anti-TB treatment initiation. Several patients had prior anti-TB treatment history before admission, which may induce persistent changes in T cell subsets and represent an unbalanced confounder within our retrospective single-center cohort. Finally, the MR analysis results lost statistical significance after strict Bonferroni multiple testing correction, so we cannot draw robust genetic causal conclusions, and more multi-ethnic MR research is required in follow-up work.

## 5 Conclusion

In conclusion, our study demonstrated that the peripheral percentage of CD4^+^CD8^+^ DP T cells in leukocytes is significantly reduced in patients with active TB, and this reduction is inversely correlated with disease severity and clinical prognosis. These findings provide novel insights into the immunopathogenesis of active TB and highlight DP T cells as promising biomarkers and potential immunotherapeutic targets for TB. Future studies are warranted to validate these findings in larger multicenter cohorts and further elucidate the functional mechanisms of DP T cells, which may facilitate clinical risk stratification and individualized management for TB patients.

## Data Availability

GWAS datasets were obtained from the IEU Open GWAS Project database. The accession numbers for immune cell traits range from ieu-a-GCST90001391 to ieu-a-GCST90002121, and the accession number for the TB dataset is ieu-a-GCST90018892. The clinical data supporting the findings of this study are available from the corresponding author upon reasonable request.

## Supplementary material

Supplementary Figure 1 Dot plots displaying the percentage of CD4^+^CD8^+^ DP T cells among total lymphocytes across four subgroups. Participants were stratified into IGRA-positive active TB (TB_IGRA^+^), IGRA-negative active TB (TB_IGRA^-^), latent TB infection (LTBI), and IGRA-negative healthy controls (HC). Horizontal lines indicate the median value of each group. All pairwise intergroup comparisons showed no statistical significance (ns, p > 0.05).
